# Trends in out of pocket payments and catastrophic health expenditure in the Kyrgyz Republic post “Manas Taalimi” and “Den Sooluk” health reforms, 2012–2018

**DOI:** 10.1186/s12939-020-01358-2

**Published:** 2021-01-11

**Authors:** Mariia Iamshchikova, Roman Mogilevskii, Michael Nnachebe Onah

**Affiliations:** Institute of Public Policy and Administration, Graduate School of Development, University of Central Asia, Bishkek, Kyrgyzstan

**Keywords:** Out-of-pocket payments, Healthcare expenditure, Catastrophic payments, Kyrgyzstan, Health system reform

## Abstract

**Background:**

Over the years, the Kyrgyz Republic has implemented health reforms that target health financing with the aim of removing financial barriers to healthcare including out-of-pocket health payments (OOPPs). This study examines the trends in OOPPs, and the incidence of catastrophic health expenditure (CHE) post the “Manas Taalimi” and “Den Sooluk” health reforms.

**Methods:**

We used data from the Kyrgyzstan Integrated Household Surveys (2012–2018). Population-weighted descriptive statistics were used to examine the trends in OOPPs and CHE at three thresholds; 10 percent of total household consumption expenditure (Cata10), 25 percent of total household consumption expenditure (Cata25) and 40 percent of total household non-food consumption expenditure (Cata40). Panel and cross-sectional logistic regression with marginal effects were used to examine the predictors of Cata10 and Cata40.

**Findings:**

Between 2012 and 2018, OOPPs increased by about US $6 and inpatient costs placed the highest cost burden on users (US $13.6), followed by self-treatment (US $10.7), and outpatient costs (US $9). Medication continues to predominantly drive inpatient, outpatient, and self-treatment OOPPs. About 0.378 to 2.084 million people (6 – 33 percent) of the population incurred catastrophic health expenditure at the three thresholds between 2012 and 2018. Residing in households headed by a widowed or single head, or residing in rural regions, increases the likelihood of incurring catastrophic health expenditure.

**Conclusions:**

The initial gains in the reduction of OOPPs and catastrophic health expenditure appear to gradually erode since costs continue to increase after an initial decline and catastrophic health expenditure continues to rise unabated. This implies that households are increasingly incurring economic hardship from seeking healthcare. Considering that this could result to forgone expenditure on essential items including food and education, efforts should target the sustainability of these health reforms to maintain and grow the reduction of catastrophic health payments and its dire consequences.

## Introduction

One of the principal goals of health reforms especially in developing countries is to ensure that households and individuals do not experience financial hardship from accessing and utilising healthcare [1]. This is based on the principle that healthcare is a human right and financial barriers are a significant impediment to healthcare access especially for the most vulnerable (the poor and sick) [2]. A key element of health reforms that aim to protect users from financial hardship is the move from direct payment for healthcare to universal health coverage where risk and financial resources are pooled for efficient healthcare access and utilisation [3]. The emphasis on financial protection to reduce or remove out- of- pocket payments (OOPPs) and catastrophic health expenditure (CHE) is established in its inclusion in the Sustainable Development Goals (SDGs) [4].

The financial protection argument stems from existing and overwhelming evidence of the catastrophic nature of direct healthcare payments in the form of OOPPs at the point of use [5–8]. Since health and healthcare are considered human rights and the demand for healthcare is largely inelastic to price fluctuations [9], households employ numerous strategies to finance healthcare and cope with the economic hardship resulting from healthcare expenditures [10, 11]. These coping strategies can include foregoing household expenditure on other basic needs including food and education, and borrowing and drawing from savings [12–15]. For households who live close to poverty thresholds, the catastrophic and impoverishing effect of healthcare expenditures can tip such households over the threshold and for those already living in poverty, healthcare expenditures can potentially deepen poverty rates [16, 17].

The Kyrgyz Republic like other Central Asian countries inherited a health system from the former Soviet Union characterised by universal health coverage and user-free health access although health systems were centralised which resulted in high bureaucratic costs and inefficiencies [18]. With the collapse of the Soviet Union and the withdrawal of economic links established during the Soviet era, most of the Central Asian countries experienced a significant reversal in economic growth [19]. This resulted in difficulties for public funds to manage social and health services and user fees were introduced [20]. Thereafter, the burden of access to services, including healthcare, was falling more on the population and more people were being pushed into poverty from accessing services including healthcare [20]. Public spending on healthcare as a percentage of gross domestic product (GDP) dropped from over 6 percent in 1994 to about 2 percent in 2004, and it was estimated that per capita government expenditure on health in 2003 was just US$66 purchasing power parity [19, 21]. Although the economy experienced important positive growths and increases in GDP in the late 1990s, recovery has been slow and funding for healthcare has suffered [22].

The Kyrgyz Republic has made significant progress to date in improving the living conditions of its populace. Poverty rates have declined to about 23 percent [23] and important reforms have been enacted to improve healthcare delivery and to also protect users from financial barriers to healthcare access and utilisation [24]. A chronology of health reforms in the Kyrgyz Republic adapted from Meimanaliev et al. [25] and Falkingham et al. [26] can be found in Table 1. In the period covering 1996–2006, the National Health Care Reform Program “Manas” was developed with the support of the World Health Organization with the primary aim of unbundling the Soviet-era health system in the Kyrgyz Republic [18]. The main features of this health reform were the creation of an infrastructure that corresponds to population needs in medical care and financial resources, the decentralization of management and enhancement of administrative and financial autonomy of health organisations, pooling of health funds, outcome-based provider payment mechanism, and the split of the health sector into providers and purchaser of healthcare services. The “Manas” health reform faced important health financing challenges. Households which utilized health services at outpatient and/or inpatient levels experienced heavy financial burden caused by persistence of informal payments and high level of co-payment, and low public health spending as a share of GDP lingered.

**Table 1:** Chronology of events and health reforms in the Kyrgyz Republic

Date	Event
1993	Introduction of user fees
March 1994	Memorandum of Understanding between WHO Regional office for Europe and the Ministry of Health of the Kyrgyz Republic to undertake the MANAS Health Care Reform Programme.
August 1994	National Health Policy approved by the government.
Nov 1996	Government approves MANAS Health Care Reform Programme. World Bank funded Health Project (1996–2000) started in Kyrgyzstan (Bishkek and Chui oblasts).
Jan 1997	Introduction of the mandatory health insurance (MHIF) system in Kyrgyzstan.
July 1997	MHIF introduces case-based payment to hospitals.
1977–1998	Rolling out of primary health care reforms to Chui, Jalal-Abad and Osh oblasts and Bishkek
June 1998	Introduction of partial fundholding in 14 Family Group Practices (FGPs) in Karakol city, Issyk-Kul oblast.
Nov 1998 – March 1999	FGPs enrolment campaign in Chui oblast and Bishkek.
Jan 1999	Introduction of capitation payment to FGPs in Bishkek.
April 1999	About 55 hospitals and 290 FGPs enter into contracts with the MHIF
Jan 2001	Government decree on Introduction of a New Health care Financing Mechanism in Health facilities of Kyrgyzstan since 2001
Feb 2002	Government decree on Provision of Health Care to Citizens of Kyrgyzstan under the State Benefits Package since 2002.
March 2002	Naryn and Talas oblasts join the single payer system
March 2003	Batken, Jalal-Abad and Osh oblasts join the single payer system.
Nov 2003	Republican facilities join the single payer system
July 2004	Law on the Single Payer System in Health Care Financing in the Kyrgyz Republic.
Feb 2006	Government approves ‘Manas Taalimi’ Health Care Reform Programme 2006–2010.
Feb 2012	National Health Reform Program “Den Sooluk” 2012–2016.
Dec 2018	The Program of the Kyrgyz Republic Government on Public Health Protection and Health Care System Development for 2019–2030 “Healthy Person - Prosperous Country”

Updated from Falkingham et al. [26] and Meimanaliev et al. [25]

### The “Manas Taalimi” health reform

The “Manas Taalimi” health reform (2006–2010) was introduced to further the progress and achievements recorded by the “Manas” health reform. An evaluation of the impact of the “Manas Taalimi” reform was performed in 2011 and health financing achievements included: (1) the consistent annual increase in health expenditures from 10 percent to 13 percent of total government expenditure; (2) the establishment of the Department of Public Health in the Ministry of Health with the goal to provide comprehensive coordination of the healthcare services and their integration with other health programs; and (3) a wage increase for healthcare workers to redress the continued increase in informal payments which was associated with low wages [27]. However, some health system issues persisted. There was a need for a stronger focus on investments that could change population health behaviour and clinical practice in order to improve the efficiency of key health interventions, the increased outflow of human resources has adversely affected both the access to healthcare and its quality, especially for vulnerable populations in remote rural areas, incomplete definition of roles and responsibilities and limited management autonomy of healthcare providers have generated a governance challenge, and adequate funding remains critical for maximizing population coverage with cost-effective healthcare services due to year of reduced funding. The “Den Sooluk” health reform was proposed to consolidate the “Manas Taalimi” health reform successes and to address identified reform gaps.

### The “Den Sooluk” health reform

The “Den Sooluk” (2012–2016) health reform is considered a logical continuation of the “Manas Taalimi” health reform with a foundation laid by deep analysis of results, problems and experience obtained during previous “Manas” and “Manas Taalimi” years and aimed at maintenance and protection of population health which would contribute significantly to poverty reduction [28]. Key strengths of the health reform include: (1) the reform proposals to match the identified health and health systems problems in a balanced way, (2) the program aims at social health protection (universal coverage, fairness in financing, equal access to services and prevention of impoverishment using international best practices), (3) the coordination, implementation and management arrangements are based on sound principles and make organisational sense, (4) the need for solving health human resources in general and for strengthening management and supervision capacity in particular are acknowledged, and (5) financial management and procurement policy and standards are adequate. An assessment of the performance of the health reform is underway. It is important to note that a review of the health reforms in the Central Asian region suggests that among the five countries, the Kyrgyz Republic has undergone the broadest, most sustained, and most successful health sector reform in the region [29].

This paper examines the trends and changes in OOPPs and the incidence of CHEs post the “Manas Taalimi” and “Den Sooluk” health reforms using data from 2012 to 2018. Although other health reforms have continued since the end of the “Den Sooluk” health reform and there might have been other non-health reform interventions which could have influenced household health expenditures, this paper focuses on a key objective of “Manas Taalimi” and “Den Sooluk” which was the protection of healthcare users from economic hardship from seeking healthcare.

### Data, empirical specification and variables

#### Data

We used data from the Kyrgyzstan Integrated Household Survey (KIHS) conducted by the National Statistical Committee (NSC). The KIHS is a national representative rotational panel household survey where approximately 25 percent of sampled households are replaced every year, and trends in important indicators, including consumption-based poverty, are examined every quarter [30]. On average, 5000 households with 22,000 individuals who were randomly selected by strata are surveyed per each round of data. The survey contains a module on household food and non-food consumption expenditure and on OOPPs for healthcare utilisation, and OOPPs data are collected for inpatient, outpatient, and self-treatment healthcare expenditures. Our analyses focused on the 2012–2018 data rounds in line with our research objective and also since the sample methodology and questionnaire design changed post 2010, this limits data comparability [22].

#### Out- of- pocket payments

For each category of healthcare expenditure (inpatients, outpatients, and self-treatment), we disaggregated our analysis to examine the components of health spending hence; for inpatient care, costs were aggregated under medications, hospitalisation, monetary value of gifts and kinds, and “others”. For outpatient care, costs were aggregated under medications, monetary value of gifts and kinds, and “others”, and for self-treatment, costs were aggregated under medications and “others”. Gifts and kinds were considered as an important cost component of OOPPs since the Kyrgyz Republic like other old Soviet Union countries inherited the tradition of presenting monetary and/or in-kind gifts to health providers and caregivers [19, 26, 31]. These gifts and in-kind payments are informal payments made to healthcare providers by healthcare users to reflect their appreciation of the services rendered however, this form of payment are indirectly expected by healthcare providers. The “other” category of health spending included costs incurred for medical supplies, diagnostic and lab services, and for inpatient care, costs also included payments to physicians, surgeons, and other hospitalisation supplies. Falkingham et al. [26] argued that they could not separate formal and informal (gifts and kinds) payments based on the likelihood that some enumerators could have been unclear whether ‘charges’ demanded by medical personnel prior to consultation were ‘official’ or not. We retained the distinction between formal and gifts and kinds (informal) payments in the study since these payments have persisted over the years and experts with local understanding of healthcare payments in the Kyrgyz Republic observes that these payment categories are clear and usually subtly expected, especially in the public health system (authors’ personal correspondence).

#### Catastrophic health expenditure

To estimate CHE, we used two approaches based on OOPPs as a share of total household consumption expenditure (food and non-food), and OOPPs as a share of total household non-food expenditure. These two approaches utilised household expenditure net of healthcare payments.

For CHE based on total consumption, we used two thresholds at 10 percent (Cata10) and 25 percent (Cata25) of total household consumption expenditure. These thresholds, called the budget share threshold, were used because they are the official Sustainable Development Goals (SDGs) thresholds for estimating CHE (SDG, 2018). A review of studies that have estimated CHE by Wagstaff et al. [5] found that in general, the budget share approach (i.e. total consumption expenditure) was the second most popular method for estimating households’ ability-to-pay for healthcare and 29 percent of studies have applied this methodology. However, in economics journals, total expenditure was the most frequently used methodology (49 percent). The thresholds of 10 percent and 25 percent were chosen since they are the most used threshold in studies that have used this approach to estimate CHE [5].

When estimating CHE as a share of household non-food expenditure, we used the threshold of 40 percent (Cata40). This approach is in line with the methodology of adjusting analysis to reflect household capacity to pay by subtracting household food expenditure or an allowance from total consumption as suggested by Xu et al. [6]. This is based on the argument that food expenditure is non-discretional and hence does not reflect household capacity to pay for expenditures after spending on food consumption [32]. Although in general, this methodology is the most popular approach to estimating CHE (31 percent of studies), in economics journals, it is the least popular approach (13 percent of studies) [5]. The threshold of 40 percent was chosen since it is the most used threshold in studies that have used this approach to estimate CHE [5].

To collect household consumption (food and non-food) and health expenditures, households kept a daily expenditure diary where costs were collected every quarter by enumerators. The frequency of data collection and diary keeping helped to limit the effect of recall bias on expenditures [30]. Diaries were examined by enumerators every quarter and follow-up questions were administered where missing or incomplete information were observed. The costs of non-food and health expenditures were aggregated per month and food expenditures were aggregated per 14 days. We extrapolated monthly food expenditure by multiplying biweekly expenditures by two. Costs were converted to 2020 US dollars [33].

#### Predictors of catastrophic health expenditure

To estimate the sociodemographic and economic characteristics that predict CHE at household and individual levels, we developed two regression models using Cata10 and Cata40 as outcome variables. We estimated only two models since the two approaches to estimating CHE use different denominators (total vs. non-food consumption expenditures) and hence, different sociodemographic and economics factors might predict CHEs differently at these thresholds.

#### Outcome variables

The two outcome variables (Cata10 and Cata40) were estimated as binary outcomes with “1” indicating catastrophic health expenditure and “0” indicating no catastrophic health expenditure.

#### Predictor variables

The KIHS collects limited socioeconomic and demographic information from respondents hence, we performed an exhaustive screening of available predictor variables in the surveys and included identified variables in the regression models.

##### Age of household members

 The ages of sick household members were grouped into five categories and were used as predictor variables based on the hypothesis that the ages of different household members might predict CHEs differently.

##### Marital status

The marital status of the household head was estimated as a categorical variable and households were grouped under the following categories: legally married and civil unions, divorced and separated, widowed, and single. This predictor variable was used based on the hypothesis that marital status of household heads might influence household socioeconomic status and CHE.

##### Sex of household members

The sex of sick household members was estimated as a predictor variable based on the assumption that sex might predict CHE differently.

##### Location

The location of households was used as a predictor variable based on the hypothesis that urban and rural households might incur CHE differently at the established thresholds.

##### Consumption expenditure quantile

This variable was developed to categorise households into different socioeconomic quantiles based on household spending adjusted for household size. Consumption spending has been used in numerous surveys and studies to estimate household poverty levels among other indicators [34]. This predictor variable was chosen based on the hypothesis that households that belong to different quantiles might incur CHE differently. Consumption expenditure included all household routine spending including utilities, rent, food, entertainment, clothing etc., but excluded once-off-payments including education.

##### Regions (oblasts)

This was included as a predictor variable based on the hypothesis that CHE might be incurred differently based on the region in which a household resides. There are seven regions and two administrative centres in the Kyrgyz Republic. Since a new administrative centre was added in the surveys post 2012 and due to changes in coding system in 2018, regions were excluded in the analyses for these two years. We chose Bishkek (an administrative centre) as the reference category in the regression analyses since it is the richest and most urban city in the Kyrgyz Republic. Hence, there are a broader variety of healthcare providers which might have an influence on OPPs and catastrophic healthcare expenditures.

#### Analysis

The data were analysed using Stata 15.1 statistical software [35]. Univariate and bivariate descriptive analyses were used to summarize the data characteristics and to examine trends in OOPPs. Univariate and bivariate analyses were population-weighted to ensure that the estimated could be extrapolated from the Kyrgyz Republic population. To achieve this, household sample weights were divided by household size. To examine the predictors of CHE, population-average estimator panel logistic regression analysis was specified, and marginal effects are reported. Population-average estimator panel regression was used since we believe that this approach provides a more useful approximation of observed association as recommended by Hubbard et al. [36]. Since the panel was rotational and hence unbalanced, we conducted the panel regression on the pooled data while each year was also analysed as cross-sectional surveys where marginal effects of logistic regressions were examined. We used this approach to examine if there were any important changes in the predictors of CHE due to the annual change in data composition. All regression analyses were population-weighted, and significance was established at 95 percent and 99 percent confidence intervals.

## Results

The description of the sample statistics is contained in Table 2. On average, household size was five, 65 percent of households were in rural areas, and approximately 62 percent of household heads were married. The proportion of households who belonged to the lowest quintile declined from about 38 percent in 2012 to nine percent in 2018. The proportion of households who belonged to the highest consumption expenditure quintile increased from 10 percent in 2012 to 32 percent in 2018. Monthly food consumption expenditure was around 4000 Soms (US $59) between 2012 and 2014 and 6000 Soms (US $87) between 2015 and 2018.

**Table 2:** Sample characteristics (percentages unless otherwise specified)

	2012	2013	2014	2015	2016	2017	2018
*Age group*							
≤ 10	17	19	25	20	20	20	20
11–20	19	17	18	16	15	15	15
21–30	11	13	13	13	12	12	11
31–40	11	11	12	11	11	11	11
41–50	14	13	12	13	13	12	12
51–60	14	13	11	12	14	15	15
> 60	12	12	8	12	12	13	12
Female	53	52	52	53	52	54	54
Rural	64	67	64	67	72	64	63
Household size (mean)	5	5	5	5	5	5	5
*Marital status*							
Married	57	62	62	62	63	65	65
Divorced/Separated	5	5	5	5	5	5	5
Widow	10	9	10	10	10	10	10
Single	28	24	24	23	23	20	20
*Consumption expenditure quantile*							
Q1 (Lowest)	38	32	20	17	13	12	9
Q2	22	23	21	20	20	18	16
Q3	17	17	21	22	22	21	19
Q4	13	15	20	21	23	24	24
Q5 (Highest)	10	13	18	19	22	25	32
*Household non-food expenditure*							
Monthly average (Soms)^a^ [median]	4467 [3369]	4674 [3542]	6395 [5305]	4644 [3320]	4735 [3458]	4567 [2939]	3984 [2410]
Utilities	35	23	20	37	36	40	31
Fuel	65	66	62	79	73	81	81

^a^households could pay for utilities, rent, and/or fuel in a month

Table 3 provides information on healthcare costs and components. On average, 40 percent of households reported a member seeking healthcare in the past month and health seeking was lowest in 2016 at 33 percent. For those that sought care, healthcare costs averaged around 1817 Soms (US $26) with highest cost recorded in 2018 (2311 Soms [US $33]). Self-treatment was the most frequent type of healthcare sought by households (78 percent), 18 percent sought outpatient care, and about 8 percent sought inpatient care. In actual spending, out- of- pocket payments remained the same at about 1862 Soms (US $27) between 2012 and 2014 and increased to about 426 Soms (US $6) by 2018. On average, outpatient costs increased from 480 Soms (US $7) to 633 Soms (US $9), inpatient costs increased from 519 Soms (US $7.5) to 940 Soms (US $13.6), and self-treatment costs increased from 257 Soms (US $3.7) to 737 Soms (US $10.7) between 2012 and 2018.

**Table 3:** Healthcare cost in the past month (percentages unless otherwise specified)

	2012	2013	2014	2015	2016	2017	2018
Sought care	41	45	41	40	33	45	49
Outpatient care (% sought care)	16	15	17	19	20	23	18
Inpatient care (% of sought care)	6	7	10	9	8	8	7
Self-treatment (% of sought care)	81	82	79	76	76	73	79
Avg healthcare expenditure per last month (Soms) [median]^a^	1885 [220]	1281 [310]	1821 [397]	2136 [400]	1795 [313]	2096 [425]	2311 [589]
Outpatient cost (Soms)	480	448	583	626	630	785	633
Inpatient cost (Soms)	519	549	928	1147	815	880	940
Self-treatment costs (Soms)	257	283	308	362	349	430	737

^a^Household could seek healthcare from multiple sources

### Trends in out- of- pocket health expenditure

**Fig. 1:**
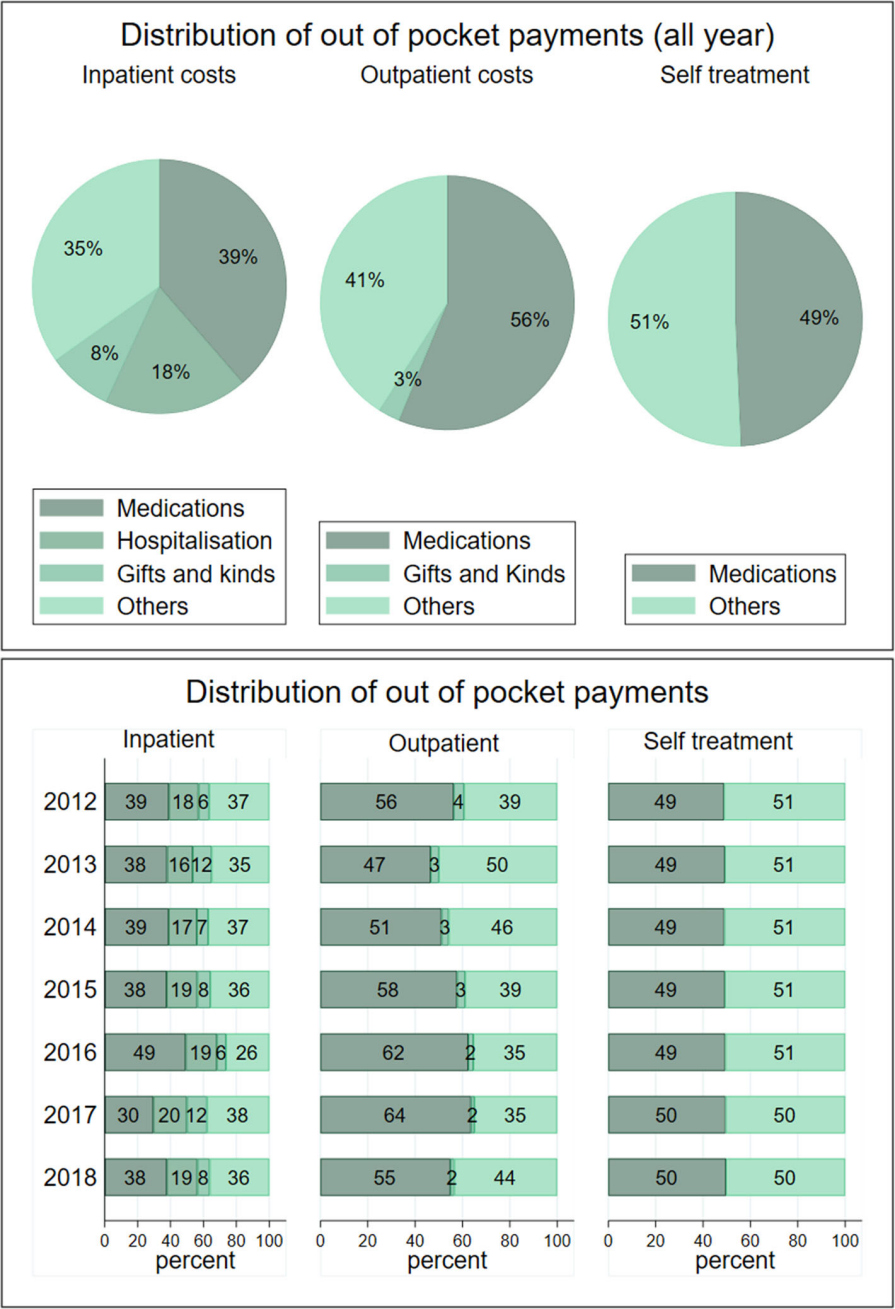
Components of out of pocket health expenditures

For households which sought inpatient care, the cost of medication was the highest driver of OOPPs (39 percent), hospitalisation costs were 18 percent, and other costs including medical supplies and payment of physicians contributed 35 percent (Fig. 1). Households, which sought inpatient care incurred a substantial cost through payments for gifts and kinds to healthcare providers as this represented 8 percent of total inpatient OOPPs. While the share of inpatient OOPPs attributed to medication costs declined over the years, the share of hospitalisation costs has increased. Medication costs as a share of inpatient OOPPs was about 39 percent between 2012 and 2015, increased to 49 percent in 2016 and declined to 30 percent and 38 percent in 2017 and 2018, respectively. Hospitalisation costs declined from 18 percent in 2012 to about 16 percent in 2013 and 2014 and increased to about 20 percent in 2017 and 2018. Payments for gifts and kinds as a share of inpatient OOPPs increased from 7 percent in 2012 to 12 percent in 2013, declined to about 8 percent in 2014 to 2016, and then increased to 12 percent and 8 percent in 2017 and 2018.

**Fig. 2:**
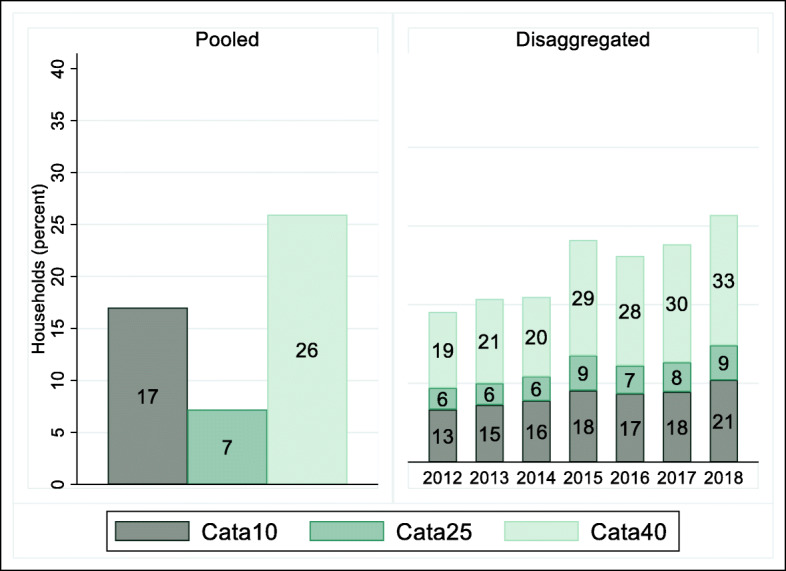
Catastrophic health expenditure at different thresholds

For households which incurred outpatient OOPPs, health expenditure initially declined from 56 percent in 2012 to 47 percent in 2013. Thereafter, there was a steady increase in medications costs as the dominant diver of outpatients OOPPs to about 55 percent in 2018 ((Fig. 2). Payments as gifts and kinds as a share of outpatients OOPPs have marginally declined from 4 percent in 2012 to 3 percent in 2015 and 2 percent in 2018. Other OOPPs, including payments for diagnostic services and medical supply as a share of outpatient OOPPs have remained the same over the years at about 40 percent. Half of households which incurred OOPPs for self-treatment spent their OOPPs on medications over the years.

### Trends in catastrophic health expenditure

Figure 2 illustrates CHEs at the three thresholds. The share of households which incurred catastrophic out- of- pocket expenditure at 10 percent of household consumption expenditure (Cata10) was 17 percent and there has been an upward trend in Cata10 over the years. The incidence of Cata10 increased by about 8 percentage-point from 13 percent in 2012 to 21 percent in 2018. A similar trend was observed when the incidence of CHE was examined as 25 percent of household consumption expenditure. The share of households which incurred Cata25 was 7 percent and this increased from 6 percent in 2012 to 9 percent in 2018.

Examining the share of households that suffered CHE at 40 percent of total household non-food consumption expenditure, the incidence of CHE was 26 percent and incidence increased by about 14 percentage-points; from 19 percent in 2012 to about 33 percent in 2018.

**Fig. 3:**
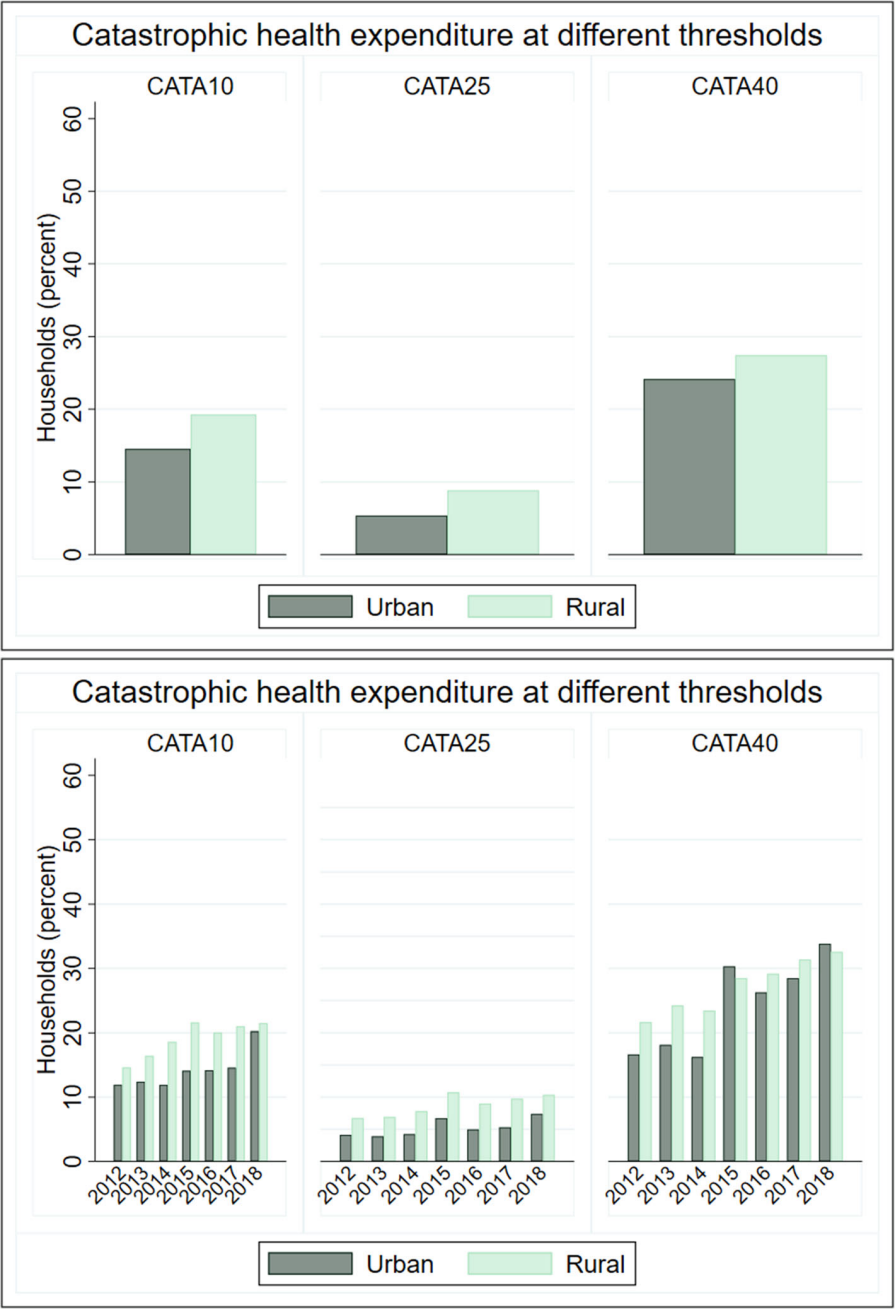
Urban-rural trends in catastrophic health expenditure

Although more rural households incurred CHE at Cata10, Cata25, and Cata40 (Fig. 3) by about 4 percentage-points, CHE increased in urban and rural areas over the years. The incidence of Cata10 increased from 12 percent in urbans areas (15 percent in rural) in 2012 to 20 percent (21 percent in rural) in 2018. The incidence of Cata25 increased from 4 percent in urban areas (7 percent in rural) in 2012 to 7 percent (10 percent in rural) in 2018. The incidence of Cata40 increased from 17 percent in urban areas (22 percent in rural) in 2012 to 34 percent (33 percent in rural) in 2018. This suggests that while more rural households incurred CHE at 40 percent of total non-food consumption expenditure, there was a more rapid increase in Cata40 in urban areas relative to rural areas.

**Fig. 4:**
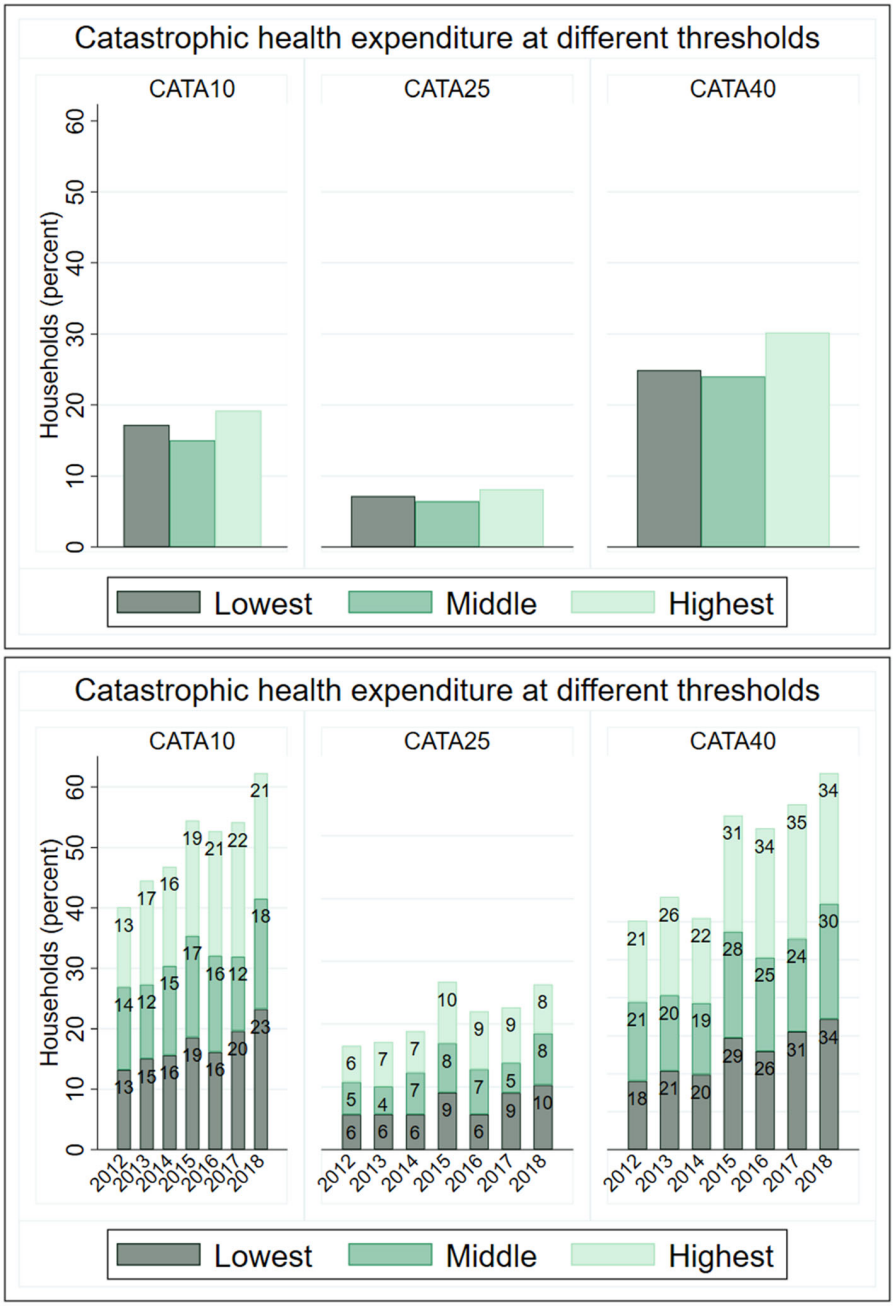
Incidence of catastrophic health expenditure by consumption expenditure quantile

While households who belonged to the highest consumption expenditure quantile incurred a higher catastrophic expenditure relative to those who belonged to the lowest quantile across the three thresholds, over the years, the incidence of catastrophic expenditure increased more rapidly for households who belonged to the lowest quantile (Fig. 3). In 2012, equal proportion of households who belonged to the lowest and highest consumption quantile incurred Cata10 (13 percent) and by 2018, the incidence has increased to 23 percent for the lowest quantile households and 22 percent for the highest. In 2012, equal proportion of households who belonged to the lowest and highest total consumption expenditure quantiles incurred Cata25 (6 percent) and by 2018, the incidence increased to 10 percent of households who belonged to the lowest quantile and 8 percent for those who belonged to the highest quantile). Similarly, the incidence of Cata40 was higher among households who belonged to the highest consumption expenditure quantile in 2012 by about 5 percentage-points, by 2018, equal proportion of households who belonged to the lowest and highest consumption expenditure quantile incurred CHE at Cata40 (34 percent) (Fig. 4). These findings suggest that CHE at different thresholds have increased over the years albeit in varying magnitude with households who belonged to the lowest quantiles experiencing more growth.

**Fig. 5:**
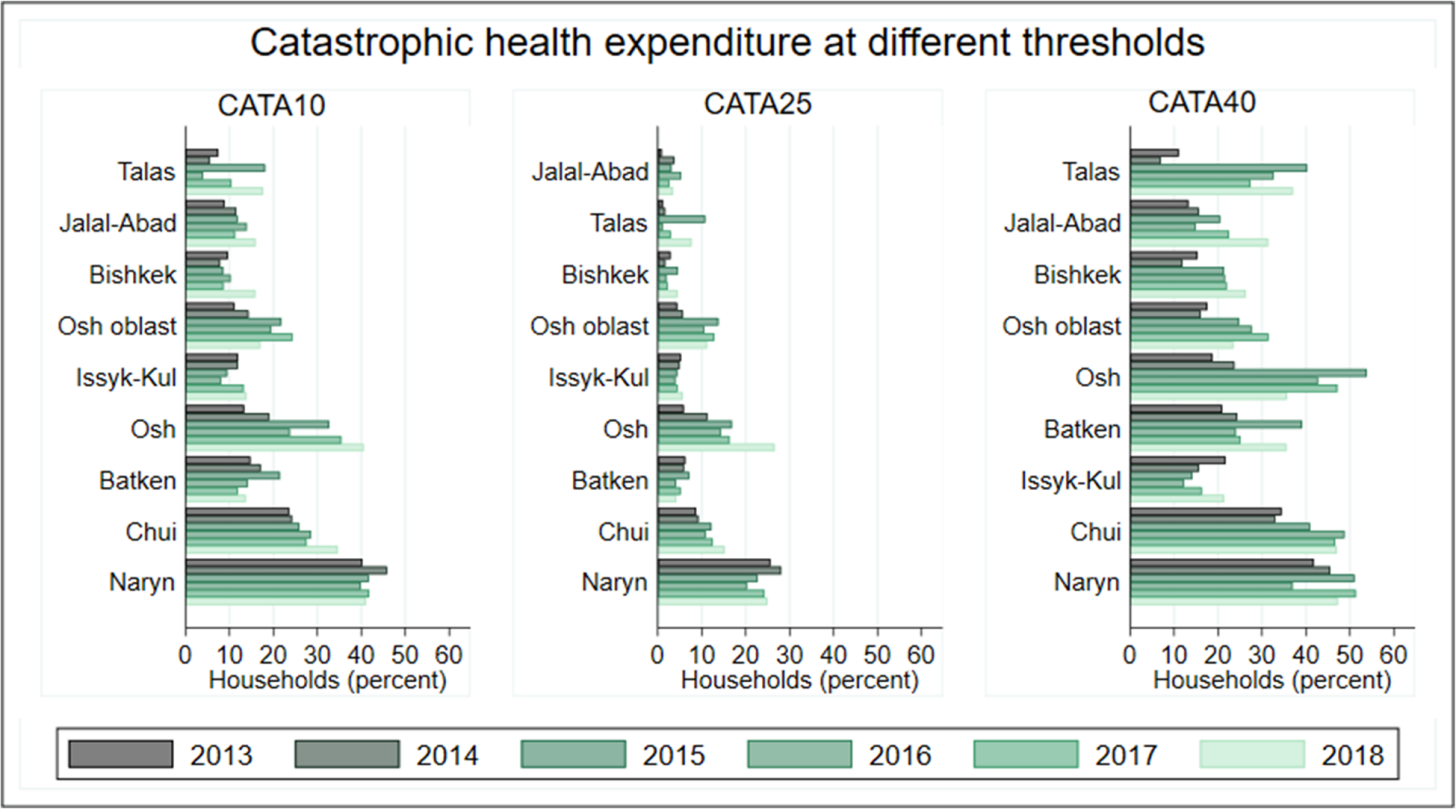
Incidence of catastrophic health expenditure by region

The incidence of CHE was most concentrated among households in Naryn, Chui, Batken, and Osh regions while Bishkek, Jalal-Abad, and Talas regions recorded the least incidence at the three thresholds (Fig. 5). Chui region recorded a 10 percentage-point increase in the incidence of Cata10 from 2013 to 2018, Osh recorded a 23 percent-point increase while Naryn region recorded a 2 percent-point increase in Cata10. This suggests that Cata10 has remained high in Naryn but have recorded the most exponential increase in Osh region. In Naryn region, the incidence of Cata25 decreased marginally from 22 percent in 2013 to 19 percent by 2018, and Cata40 increased from 35 percent in 2013 to 37 percent by 2018. In Bishkek, the incidence of Cata10 and Cata25 increased by about 6 and 2 percent between 2013 and 2018 respectively while the incidence of Cata40 increased from 15 percent in 2013 to 22 percent by 2017. Between 2013 and 2018, Talas region recorded a 4 percentage-point increase in the incidence of Cata10 and Cata25, and the incidence of Cata40 increased by 13 percentage-point.

### Predictors of catastrophic health expenditure

The predictors of CHE are presented in Tables 4 and 5, and only significant associations are present in text.

**Table 4:** Marginal effects of logistic regression for determinants of Cata10

Variables	Pooled	2012	2013	2014	2015	2016	2017	2018
*Age groups [Ref: 31–40 years]*								
< 10 years	0.012* (0.006)	0.014 (0.010)	0.015 (0.010)	0.018* (0.010)	0.018* (0.010)	0.016* (0.010)	0.018* (0.010)	0.019* (0.010)
11–21 years	0.017*** (0.005)	0.027*** (0.007)	0.025*** (0.007)	0.027*** (0.007)	0.027*** (0.007)	0.027*** (0.007)	0.027*** (0.007)	0.027*** (0.007)
21–30 years	-0.007 (0.004)	-0.006 (0.006)	-0.004 (0.006)	-0.002 (0.006)	-0.003 (0.006)	-0.004 (0.006)	-0.004 (0.006)	-0.003 (0.006)
41–50 years	0.013*** (0.005)	0.025*** (0.006)	0.026*** (0.006)	0.026*** (0.006)	0.026*** (0.006)	0.025*** (0.006)	0.025*** (0.006)	0.025*** (0.006)
51–60 years	0.041*** (0.005)	0.069*** (0.007)	0.072*** (0.007)	0.072*** (0.007)	0.072*** (0.007)	0.072*** (0.007)	0.072*** (0.007)	0.071*** (0.007)
> 60 years	0.003 (0.002)	0.007** (0.004)	0.007* (0.004)	0.007* (0.004)	0.007* (0.004)	0.007* (0.004)	0.007* (0.004)	0.007* (0.004)
*Marital status [Ref: Married]*								
Divorced/Separated	0.010 (0.007)	0.017* (0.010)	0.018* (0.010)	0.016* (0.010)	0.016* (0.010)	0.018* (0.010)	0.014 (0.009)	0.016 (0.010)
Widow(er)	0.021*** (0.005)	0.030*** (0.006)	0.031*** (0.006)	0.031*** (0.006)	0.031*** (0.006)	0.031*** (0.006)	0.031*** (0.006)	0.032*** (0.006)
Single	0.017*** (0.005)	0.025*** (0.008)	0.023*** (0.008)	0.024*** (0.008)	0.024*** (0.008)	0.023*** (0.008)	0.024*** (0.008)	0.025*** (0.008)
Rural location	0.012** (0.005)	0.020*** (0.004)	0.018*** (0.004)	0.019*** (0.004)	0.018*** (0.004)	0.017*** (0.004)	0.016*** (0.004)	0.017*** (0.004)
*Consumption quantile [Ref: Highest]*								
Lowest	0.008** (0.004)	-0.030*** (0.005)	-0.025*** (0.005)	-0.021*** (0.005)	-0.019*** (0.005)	-0.020*** (0.005)	-0.021*** (0.005)	-0.020*** (0.005)
Middle	-0.009** (0.004)	-0.038*** (0.005)	-0.035*** (0.005)	-0.033*** (0.005)	-0.033*** (0.005)	-0.034*** (0.005)	-0.034*** (0.005)	-0.034*** (0.005)
*Regions (oblasts) [Ref: Bishkek]*								
Issyk-Kul	0.021** (0.009)		0.018*** (0.006)	0.020*** (0.006)	0.020*** (0.006)	0.020*** (0.006)	0.020*** (0.006)	
Jalal-Abad	0.011 (0.009)		0.013** (0.006)	0.015** (0.006)	0.016*** (0.006)	0.015*** (0.006)	0.013** (0.006)	
Naryn	0.275*** (0.012)		0.290*** (0.009)	0.286*** (0.009)	0.290*** (0.009)	0.285*** (0.009)	0.283*** (0.009)	
Batken	0.037*** (0.010)		0.058*** (0.007)	0.057*** (0.006)	0.059*** (0.007)	0.059*** (0.006)	0.055*** (0.006)	
Osh	0.060*** (0.010)		0.062*** (0.007)	0.063*** (0.007)	0.068*** (0.007)	0.069*** (0.007)	0.065*** (0.007)	
Talas	-0.020** (0.009)		-0.007 (0.006)	-0.006 (0.006)	-0.003 (0.006)	-0.005 (0.006)	-0.005 (0.006)	
Chui	0.131*** (0.010)		0.142*** (0.007)	0.142*** (0.007)	0.144*** (0.007)	0.143*** (0.007)	0.138*** (0.007)	
Osh city	0.165*** (0.017)		0.190*** (0.010)	0.188*** (0.010)	0.193*** (0.010)	0.194*** (0.010)	0.184*** (0.009)	

Standard errors in parentheses; *** *p* < 0.01, ** *p* < 0.05, * *p* < 0.1

**Table 5:** Marginal effects of logistic regression for the determinants of Cata40

Variables	Pooled	2012	2013	2014	2015	2016	2017	2018
*Age groups [Ref: 31–40 years]*								
< 10 years	-0.008 (0.008)	-0.014 (0.011)	-0.013 (0.011)	-0.009 (0.011)	-0.009 (0.011)	-0.011(0.011)	-0.008 (0.011)	-0.014 (0.011)
11–21 years	0.015*** (0.006)	0.017** (0.008)	0.015* (0.008)	0.018** (0.008)	0.018** (0.008)	0.016** (0.008)	0.017** (0.008)	0.013 (0.009)
21–30 years	-0.014** (0.006)	-0.010 (0.007)	-0.006 (0.008)	-0.003 (0.008)	-0.004 (0.008)	-0.006 (0.007)	-0.005 (0.007)	-0.009 (0.008)
41–50 years	0.017*** (0.006)	0.025*** (0.008)	0.027*** (0.008)	0.028*** (0.008)	0.028*** (0.008)	0.027*** (0.008)	0.027*** (0.008)	0.019** (0.008)
51–60 years	0.055*** (0.007)	0.102*** (0.009)	0.107*** (0.009)	0.107*** (0.009)	0.107*** (0.009)	0.107*** (0.009)	0.108*** (0.009)	0.093*** (0.009)
> 60 years	0.005 (0.003)	0.014*** (0.005)	0.013*** (0.005)	0.014*** (0.005)	0.013*** (0.005)	0.013*** (0.005)	0.014*** (0.005)	0.014*** (0.005)
*Marital status [Ref: Married]*								
Divorced/Separated	0.024*** (0.008)	0.045*** (0.011)	0.047*** (0.011)	0.045***(0.011)	0.045*** (0.011)	0.046*** (0.011)	0.041*** (0.011)	0.046*** (0.012)
Widow(er)	-0.014** (0.006)	-0.014* (0.008)	-0.014* (0.008)	-0.015* (0.008)	-0.015* (0.008)	-0.014* (0.008)	-0.014* (0.008)	-0.015* (0.008)
Single	-0.007 (0.006)	-0.006 (0.009)	-0.002 (0.009)	-0.003 (0.009)	-0.003 (0.009)	-0.002 (0.009)	-0.004 (0.009)	-0.003 (0.009)
Rural location	-0.009 (0.006)	-0.002 (0.005)	-0.006 (0.005)	-0.004 (0.005)	-0.006 (0.005)	-0.008* (0.005)	-0.009* (0.005)	-0.008* (0.005)
*Consumption quantile [Ref: Highest*								
Lowest	-0.080*** (0.005)	-0.116*** (0.006)	-0.111*** (0.006)	-0.105*** (0.006)	-0.102*** (0.006)	-0.102*** (0.006)	-0.104*** (0.006)	-0.100*** (0.006)
Middle	-0.063*** (0.005)	-0.094*** (0.006)	-0.092*** (0.006)	-0.089*** (0.006)	-0.088*** (0.006)	-0.089*** (0.006)	-0.089*** (0.006)	-0.088*** (0.006)
*Regions (oblasts) [Ref: Bishkek]*								
Issyk-Kul	-0.093*** (0.012)		-0.094*** (0.008)	-0.092*** (0.008)	-0.091*** (0.008)	-0.089*** (0.008)	-0.091*** (0.008)	
Jalal-Abad	-0.024** (0.012)		-0.048*** (0.008)	-0.046*** (0.008)	-0.044*** (0.008)	-0.044*** (0.008)	-0.048*** (0.008)	
Naryn	0.179*** (0.014)		0.181*** (0.010)	0.177*** (0.010)	0.182*** (0.010)	0.179*** (0.010)	0.174*** (0.010)	
Batken	-0.004 (0.013)		0.012 (0.009)	0.010 (0.009)	0.014 (0.009)	0.016* (0.009)	0.007 (0.009)	
Osh	-0.061*** (0.012)		-0.062*** (0.009)	-0.060*** (0.009)	-0.054*** (0.009)	-0.051*** (0.009)	-0.056*** (0.009)	
Talas	-0.043*** (0.014)		-0.044*** (0.009)	-0.041*** (0.009)	-0.035*** (0.009)	-0.036*** (0.009)	-0.039*** (0.009)	
Chui	0.108*** (0.013)		0.105*** (0.009)	0.106*** (0.009)	0.109*** (0.009)	0.109*** (0.009)	0.101*** (0.009)	
Osh city	0.151*** (0.019)		0.167*** (0.011)	0.165*** (0.011)	0.173*** (0.011)	0.176*** (0.011)	0.161*** (0.011)	

Standard errors in parentheses; *** *p *< 0.01, ** *p* < 0.05, * *p* < 0.1

Households with a sick member who was between the ages of 11–20 (1.7 percentage-point increase in likelihood), 41–50 (1.3 percentage-point increase in likelihood), and 51–60 (4.1 percentage-point increase in likelihood) were more likely to incur Cata10 relative to when a sick member was between 31 and 40 years old. Households headed by individuals who were widowed (2.1 percentage-point increase in likelihood) or single (1.7 percentage-point increase in likelihood) were more likely to incur Cata10 when they were sick and sought care relative to those headed married individuals. The magnitude of these associations increased to about 3.0 and 2.5 percentage-point respectively when analyses were disaggregated by year. Rural households with a sick member who sought care were about 1.2 percentage-point more likely to incur Cata10 relative to urban households, and the magnitude of association decreased to about 2.0 percentage-point when analyses were disaggregated.

Although the magnitude of association was small, while households who belonged to the lowest consumption expenditure quantile were more likely to incur Cata10, households who belonged to the middle quantile was less likely to incur Cata10 when a member was sick and sought care. Belonging to the lowest quantile was associated with a 0.8 percentage-point increase in the likelihood of incurring Cata10 while belonging to the middle quantile was associated with a 0.9 percentage-point decrease in the likelihood of incurring Cata10. These associations were also observed in the disaggregated analyses. Relative to the richest and most developed region in the Kyrgyz Republic (Bishkek), household who resided in Issyk-Kul, Naryn, Osh, Osh city, and Chui were more likely to incur Cata10 when a member was sick and sought care. The magnitude of the association strongest in Naryn, Chui, and Osh city regions suggesting a stronger association in these regions.

Households with a sick member who was between 11 and 20 (1.5 percentage-point increase in likelihood), 41–50 (1.7 percentage-point increase in likelihood), and 51–60 years old (5.5 percentage-point increase in likelihood) were more likely to incur Cata40 relative to when a sick member was between 31 and 40 years old. The magnitude of the significant association remained relatively the same when analyses were disaggregated (Table 5). Relative to households headed by married heads, there was a 2.4 percentage-point increase in the likelihood of incurring Cata40 when a sick member who sought care belonged to households headed by divorced or separated heads. The magnitude of this association increased to about 4.5 percentage-point when analyses were disaggregated by year. Although at varying magnitudes, households that belonged to lower and middle consumption quantiles were less likely to incur Cata40 when a household member was sick and sought care (see Table 5 for more details). Relative to richest and most developed region (Bishkek), while household who resided in Issyk-Kul, Jalal-Abad, Osh, and Talas were less likely to incur Cata40 when a member was sick and sought care, households who resided in Naryn, Chui and Osh city were more likely to incur Cata40 when a member was sick and sought care. The magnitude of the association in Naryn, Chui, and Osh city regions were stronger than those in the other regions suggesting a stronger association (see Table 5 for more details).

## Discussion

Our data suggest that the initial progress made in reducing the level of OOPPs for healthcare use and the incidence of CHE by the “Manas Taalimi” and the “Den Sooluk” health reforms is beginning to erode since costs are rising and the incidence of CHE is increasing. Although a key priority of the health reforms were to improve on social protection for healthcare utilisation and to reduce economic hardship from seeking healthcare, our data suggests that OOPPs have increased by about US $6 over the years. In 2007, Falkingham et al. [26] estimated inpatient out-of-pocket costs in the Kyrgyz Republic at US $17.1 and our study estimates that while inpatient costs reduced to US $7.5 in 2012, costs increased to US $13.6 in 2018. This suggests that although the “Den Sooluk” health reform consolidated the impact of the “Manas Taalimi” health reform in reducing out-of-pocket healthcare costs, costs are rising post health reforms. This is most significant when patients self-treat since cost rose by about US $7 between 2012 and 2018. The increase in OOPPs and incidence of CHEs can be attributed to the successful implementation of components of these health reforms since studies have suggested that when health reforms improve access to healthcare but does not adequately protect users from OOPPs, the incidence of OOPPs and CHE tend to increase [37, 38]. A review of the performance of health systems reforms in the Kyrgyz Republic from independence to 2010 by Ibraimova et. al. (2011) [24] found systemic improvements in geographic equality in health systems, improvement in the quality of healthcare delivery, and the targeting of key primary healthcare needs. Our findings suggest that perhaps there has been lapses in ensuring the sustainability of other components of the reforms consolidated by “Manas Taalimi” and “Den Sooluk” health reforms especially where it concerns financial protection from healthcare utilisation.. Evidence from China [39, 40] and Peru [41] also suggests an effect of variations in the level of implementation of health reforms that improve the quality of health systems and reduce financial barriers to health seeking on increases in OOPPs and CHEs. Within the Central-Asian region, exponential rising cost of healthcare for end-users has also been reported in Tajikistan [42] and Kazakhstan [43] due to rising OOPPs.

Medication remains the highest driver of OOPPs and while medication costs as a share of out-of-pocket inpatient costs declined over the years, as a share of outpatient costs, there has been a noticeable increase. Baschieri and Falkingham, [19] observed that in 2004, 70 percent of households which sought inpatient care in the Kyrgyz Republic paid for medications out of pocket. Examining OOPPs for inpatient care in the Kyrgyz Republic between 2001 and 2017, Falkingham et al. [26] observed a decline from about 80 percent to 57 percent for medication payments. Comparing this to our study, findings suggest that OOPPs for medication as a share of inpatient costs have further declined to 34 percent in 2018. However, it is not clear if outpatient medication costs were separated from inpatient costs in the study by Falkingham et al. [26] and perhaps our finding of a decline is due to increases in other costs including payment for specialist care as a share of OOPPs. Data from other Central Asia countries suggest that medication costs are rising. In Tajikistan, Schwarz et al. [42] observed that expenditures on medicine represent the biggest financial burden for patients accessing primary care and rose from about 77 percent in 2005 to about 84 percent in 2011. In Kazakhstan, OOPPs for medications can get as high as 67 percent of total healthcare expenditure [44]. These findings suggest that medication costs remain the major driver of out-of-pocket health expenditure in the Central Asia region.

Payments to healthcare providers as gifts and kinds (informal payments) remain an important out-of-pocket expenditure especially for households who seek inpatient care and could cost about 9 percent (and as high as 13 percent) of total inpatient cost. Comparability of our findings to previous studies in Central Asia is limited since previous studies reported difficulties in separating formal and informal payments [19, 26, 45]. Jakab et al. [46] using data from hospitalised patients in the Kyrgyz Republic found that the proportion of patients who made informal payments to medical personnel recorded an initial declined from 2001 to 2006 but increased to about 60 percent in 2013 and the mean payment could be as high as US $23 depending on the type of facility. This suggests that informal payments to medical personnel could singularly be catastrophic for inpatient healthcare users. For those who self-treat, about 50 percent of costs are spent on medication and costs have remained the same over the years. Considering the high prevalence of self-treatment, this should be an area of focus for subsequent health reforms since it is a significant driver of OOPPs. Evidence from other Central Asia countries suggest that informal payments as a share of total OOPPs continue to rise. In Tajikistan, informal payments can be as high as 20–73 percent of healthcare spending between 2005 and 2011 [42]. In other settings especially among low-and-middle-income countries, there are different varieties of informal payments; gifts and in-kind payments, informal charges for free-of-charge services, and bribes [47]. The growth in the rate of these informal payments have led to a general consensus across studies in these settings that informal payments could potentially increase OOPs and CHEs [48–50].

At the national level, about 1.073 million people incurred CHE at the 10% threshold (from 0.821 million in 2012 to 1.2 million in 2018), about 0.442 million people for Cata25 (from 0.378 million in 2012 to 0.568 million in 2018), and about 1.642 million people for Cata40 (1.2 million in 2012 to 2.084 million in 2018). The trends in the incidence of CHE suggests that healthcare expenditure is increasingly becoming regressive in the Kyrgyz Republic. Although the incidence of CHE at the three thresholds had evened-out across households irrespective of their socioeconomic status and urban/rural location in 2012, over the years, lower wealth status households (based on consumption expenditure classification) and households who reside in rural areas are beginning to disproportionately incur more CHE. A study conducted by Wagstaff et al. (2018) using earlier rounds of the KIHS data (2005 to 2011) estimated Cata10 at about 10–14 percent and Cata25 at about 1.5–3.1 percent. Comparing this finding to our study, findings indicate that CHEs have continued to increase using the SDGs thresholds post “Manas Taalimi” and “Den Sooluk” health reforms. Estimating catastrophic expenditure using 40 percent of total non-food consumption expenditure as the threshold, findings also suggest that the incidence of CHE have increased post the study conducted by Wagstaff et al. (2018) where incidence was estimated at 4–13 percent between 2005 and 2011. Further, a study conducted by the World Health Organisation estimated an initial decline from 15 percent in 2010 to 10 percent in 2012 but incidence increased to about 13 percent by 2014 [22]. Our study finding suggests that while the incidence of CHE appear to be higher when estimated as a share of household non-food expenditure, at other thresholds, the incidence of CHE has risen over the years in the Kyrgyz Republic. To summarize, between 2001 and 2007, there was a rise in OOPs and CHEs [26] which declined by 2011 [5] and our finding suggest a gradual increase by 2018. Data also shows that OOPPs is becoming more regressive as the poor and rural dwellers are incurring more CHEs. This highlights the need for the Kyrgyz government to ensure that the initial progress made in reducing OOPPs and CHEs are regained by ensuring better compliance to policy components that limit formal charges and discourage informal charges at the point of utilisation. Further, these policies should be enforced at scale to ensure equity across regions and locations.

There is limited comparable data that have examined the predictors of CHE especially in Central Asia. Although we could only examine associations and not causality, our study findings suggest that residing in households headed by a widowed or single head, or residing in rural regions (Naryn, Osh, and Chui), when sick, increases the likelihood of households incurring CHE when care is sought. While households in the lowest consumption expenditure quantile were more likely to incur Cata10 relatively to the highest quantile, they were less likely to incur Cata40. This adds to the existing evidence from other jurisdictions that suggest that perhaps food expenditure for low status households impacts on their capacity to pay for healthcare (i.e. food expenditure takes a significant chunk of their spending) [16]. These findings adds to the limited literature and provides comparable data for future studies in the Central Asia region.

Our study has several limitations. The KIHS do not collect information on the nature of ill-health and hence, we could not determine if catastrophic health payment was related to specific healthcare expenditures. The KIHS also discontinued the collection of detailed information on OOPPs including on co-payments, timing of informal payments, and type of facility utilised (private, public, etc.). Also, the KIHS do not collected more detailed economic and sociodemographic information from respondents including income, education, and sex of household head. These are important variables to include in the analysis of OOPPs and CHE.

In conclusion, the initial progress in reducing the growth in out of pocket payments and catastrophic health expenditure experienced with the introduction of the “Manas Taalimi” and “Den Sooluk” health reforms appears to be gradually eroded over the years since costs continue to increase after an initial decline and catastrophic health expenditure continues to rise unabated. The implication is that more individuals and households are incurring economic hardship from seeking healthcare. This might have a negative effect on the ability of vulnerable populations to cope with growing healthcare costs even as demand for better healthcare might have increased. While the “Den Sooluk” and “Manas Taalimi” and previous health reforms have improved access to healthcare through a more equitable distribution of services, the Kyrgyz government should ensure that compliance towards the implementation of other key components of these health reforms including the removal (or at the least; reduction) of OOPPs are sustained to protect the poor and sick from CHEs. This would ensure that such increase in utilisation of healthcare does not come with economic hardship by keeping healthcare spending at the point of use at least affordable. Although the Kyrgyz Republic has been at the forefront of health reforms in Central Asia, the sustainability of health reforms is crucial in redressing and limiting the decline in the successes achieved through significant health reforms.

## Data Availability

The data is available at request from the National Statistical Committee in Kyrgyzstan (http://www.stat.kg/en/).

## Abbreviations

GDP: Gross Domestic Product
SDG: Sustainable Development Goals
OOPPs: Out-of-pocket payments
CHE: Catastrophic health expenditure
Cata10: Catastrophic health payment at 10% of total household consumption expenditure
Cata25: Catastrophic health payment at 25% of total household consumption expenditure
Cata40: Catastrophic health payment at 40% of total household non-food consumption expenditure
KIHS: Kyrgyzstan Integrated Household Surveys
US$: United States dollars
